# Fallopian Tube Originating Ovarian Cancer in a 53-Year-Old Postmenopausal Female With Hereditary Breast Cancer (BRCA) Genes: A Case Study

**DOI:** 10.7759/cureus.23929

**Published:** 2022-04-07

**Authors:** Iana Garrick, Habiba Ahasan, Matthew Jiang, Yakubmiyer Musheyev, Farage Ftiha, Maria Levada

**Affiliations:** 1 Medicine, New York Institute of Technology College of Osteopathic Medicine (NYITCOM), Old Westbury, USA; 2 Obstetrics and Gynecology, New York Institute of Technology College of Osteopathic Medicine (NYITCOM), Old Westbury, USA

**Keywords:** serous carcinoma, bilateral salpingo-oophorectomy, brca, fallopian tube, ovarian cancer, postmenopausal

## Abstract

Ovarian cancer is the second most common gynecologic malignancy, but it is the deadliest of the gynecologic cancers. Out of 21,410 new cases of ovarian cancer in the United States in 2021, more than half were fatal. In this case study, a 53-year-old sexually active postmenopausal patient with a family history of breast cancer presented to her gynecologist for an annual exam. Given the patient’s family history and breast cancer mutations, malignancy was a concern that had to be addressed. Elective bilateral salpingo-oophorectomy of the patient revealed ovarian serous carcinoma originating from the fallopian tubes. Historically, fallopian tube carcinoma was presumed to be rare, though many high-grade serous carcinomas previously classified as advanced ovarian carcinomas are now believed to have actually originated from the fallopian tubes. This case study adds to the body of evidence that many high-grade carcinomas have fallopian tube origins. This emerging perspective of ovarian cancer’s origin provides healthcare workers and the scientific community a more complete picture of the etiologies and dissemination pattern of ovarian cancer. We hope this study will help physicians have a more extensive knowledge base of such a disease when looking for risk factors and taking care of their patients.

## Introduction

The term “ovarian cancer” is used to describe a group of cancers arising from cells of the ovaries, fallopian tubes, or peritoneum as these cancers share clinical behavior and treatment. In the United States, ovarian cancer is the second most common gynecologic malignancy, but it is the deadliest of the gynecologic cancers. Out of 21,410 new cases of ovarian cancer in the United States in 2021, 13,770 were fatal (an overwhelming mortality rate of 64.3%) [1].

The majority of primary ovarian cancers (95%) are epithelial, with the remaining being germ cell or sex cord-stromal tumors [2]. High-grade serous, low-grade serous, endometrioid, clear cell, and mucinous subtypes of epithelial ovarian cancer exist; serous is the most frequent subtype (75%) of epithelial carcinomas [2]. About 18% of epithelial ovarian cancer diagnoses are associated with germline mutations, most of which are attributable to breast cancer susceptibility gene 1 (BRCA1) and breast cancer susceptibility gene 2 (BRCA2) mutations [2]. 

In the United States, the average age of diagnosis of ovarian cancer is 63 years [3]. However, patients with hereditary ovarian cancer syndrome had a lower age at diagnosis of ovarian cancer, with the median age of diagnosis for BRCA1 and BRCA2 carriers being 54.0 and 59.5 years, respectively [4]. In fact, pathogenic mutations in BRCA1 and BRCA2 are the most powerful risk factors for breast and ovarian cancer development. Pathogenic mutations in these genes are responsible for 20% of ovarian cancer cases [1]. As a result, individuals with a personal or family history of breast, ovarian, prostate, or pancreatic cancer may benefit from a genetic risk assessment to determine their own and their family members' risk for these malignancies.

Patients with epithelial ovarian cancer (EOC) exhibited considerably greater rates of the following symptoms versus those with benign masses: increased abdominal size (64% versus 56% and 19%), bloating (70% versus 49% and 38%), and urinary tract symptoms, particularly urgency (55% versus 31% and 32%) [5]. Given enough clinical suspicion, follow-up generally involves assessment of risk factors, pelvic imaging, and surgical intervention. Furthermore, serum biomarkers such as CA 125 levels can be used to monitor disease progression. Up to 80% of patients with EOC will have an elevated CA 125 [5]. However, the diagnostic effectiveness of CA 125 is limited, especially in early-stage diseases. Furthermore, assessing this serum biomarker is primarily beneficial to postmenopausal women as the specificity is very low in premenopausal patients [6]. For EOC, combining CA 125 and the symptom index may provide better diagnostic results than either test alone [6].

An additional consideration in symptom evaluation must be made in women of postmenopausal age since chills, weight changes, and incontinence can occur in the menopausal stage. Postmenopausal bleeding is a concerning symptom and can be an important clinical indicator of various gynecological malignancies, thus patients with this sign should be screened for endometrial hyperplasia/carcinoma [7]. Screening tools include hereditary cancer syndrome questionnaires, pelvis sonograms, CT scans of the lower abdomen and pelvis, and given high suspicions, endometrial biopsy, and cytopathology analysis [6].

Laboratory tests and imaging are important but overall, EOC is a histologic diagnosis based on tissue pathology examination after surgical removal of an ovary or fallopian tube or peritoneal biopsies. Tissue or fluid acquired via image-guided biopsy, paracentesis, or thoracentesis is used less commonly to make the diagnosis [6]. Early identification of EOC is optimal to improve the prognosis by detecting the disease while it is localized. Unfortunately, at the time of diagnosis, over 80% of patients have lymph node involvement or distant metastases [3], meaning the majority of patients have stage III or greater disease [8]. As a result, the current approach to combating late-stage ovarian cancer involves an early index of suspicion via early recognition of high-risk patients, appropriate clinical studies, and treatment via surgical resection, chemotherapy, and other available means. In this case study, early diagnosis and intervention led to a successful removal and treatment of ovarian cancer.

## Case presentation

After a long set of fears and two years of physician counseling, a 53-year-old female patient with a family history of breast cancer was finally ready to obtain BRCA genetic testing. Initially, the patient presented with postmenopausal bleeding. Her last menstrual period was two years prior to the current visit (51 years). Past pap smears and mammograms were unremarkable, and an endometrial biopsy found no abnormal endometrial pathology.

Her family history was significant for a mother and older sister with early-onset breast cancer, diagnosed before 45 and 40 years old, respectively. Her sister passed away at 55 years from metastatic breast cancer. The patient has a medical history of hypertension and thyroid disorders for which she has been taking variable doses of lisinopril-HCTZ, Synthroid, vitamin D, and calcium throughout the years. The patient had previous surgeries to remove basal cell cancer of the chin and a thyroidectomy at 15 years to treat goiter.

The patient has four children: Gravida - 4, para - 4 (living), and vaginal delivery - 4. The patient smoked tobacco on and off for the past 23+ years. She consumes alcohol on occasion and is allergic to shellfish. The patient did not present with clinical symptoms (bloating, urinary incontinence, increased abdominal size, etc.), though this is not uncommon in earlier stages of ovarian cancer.

Complete blood count (CBC), blood urea nitrogen/creatinine (BUN/Cr), urinalysis, and routine blood tests were within normal limits. Mammograms found benign fibrocystic changes, and the most recent bilateral breast and abdominal sonography had no unusual findings. Chest x-ray and CT of the pelvis and abdomen were performed with no remarkable findings. The patient underwent salivary BRCAnalysis® that found a deleterious mutation of BRCA1 and a genetic variant, favoring polymorphism of BRCA2, specifically BRCA2 E2981K (9169G->A). The BRCA1 R1443X (4446C->T) mutation resulted in a premature protein truncation of BRCA1.

Given the patient’s family history and positive BRCA gene mutations, the patient underwent preventative, elective laparoscopic bilateral oophorectomy and salpingectomy with fractional dilation and endometrial curettage under general anesthesia. Gross examination of the left fallopian tube was unremarkable: multiple translucent cysts of 0.1-0.3 cm filled with clear serous fluid were found. The right fallopian tube was also grossly unremarkable and did not contain any cysts. The pathology report revealed serous carcinoma of the left and right fallopian tubes.

A 3-mm primary tumor of poorly differentiated serous carcinoma was identified in the left fallopian tube. Sectioning of the paraffin-embedded formalin-fixed specimen revealed focal hyperplasia of the tubal epithelium. Microscopic fragments of moderately differentiated, papillary serous carcinoma were found within the lumen of the right fallopian tube along with hyperplastic and dysplastic tubular epithelium.

Both the left and right ovaries were negative for any tumors: Grossly, the left ovary was pale pink and slightly convoluted, and upon sectioning, it had a 1.3-cm cyst filled with clear serous fluid, whereas the right ovary was pale pink and slightly convoluted with no cysts (Tables 1, 2). Subsequent microscopic examination showed no invasion by the fallopian ovarian serous carcinoma into the ovaries.

**Table 1:** Core findings of ovarian cancer in the cytopathology report for bilateral salpingo-oophorectomy

Sample	Size	Gross appearance	Tumor size	Histological type	Histological grade	Microscopic tumor extension	Lymph-vascular invasion	Additional pathologic findings	Pathological staging
Left fallopian tube	4.7 cm x 0.5 cm	Grossly unremarkable	3 mm	Serous carcinoma	Poorly differentiated	Fimbria	Not seen	Focal hyperplasia of tubal epithelium	pT1c
Right fallopian tube	5.7 cm x 0.4 cm	Partially attached to ovary, unremarkable	Cannot be determined (microscopic intraluminal foci)	Papillary serous carcinoma	Moderately differentiated	Microscopic fragments of papillary serous carcinoma within lumen	Not identified	Focal hyperplasia and dysplasia of tubal epithelium	

**Table 2:** Additional findings from the cytopathology report for bilateral salpingo-oophorectomy and follow-up total hysterectomy

Sample	Size	Gross appearance	Final diagnosis
Left ovary	2.5 cm x 1.5 cm x 1.5 cm	Ovary is pale pink, slightly convoluted with multiple translucent cysts, filled with serous fluid	Negative for tumor, cysts filled with clear serous fluid identified
Right ovary	2.5 cm x 1.5 cm x 1 cm	Ovary is pale pink, slightly convoluted	Negative for tumor
Endometrial curetting	NA	Hemorrhagic mucinous material	Benign squamous mucosa: scanty fragments of inactive endometrial epithelium
Pelvic washing	60 cc blood tinged fluid with large amount of material in solution	Positive for malignant cells: metastatic high-grade carcinoma	
Uterus and cervix	Uterus with cervix: 6 cm x 5 cm x 3.8 cm; endometrial cavity: 2 cm x 4.4 cm; myometrium: 1.6 cm thick	Smooth tan-gray uterine serosa, areas of thickened endometrium with the remaining endometrium having a tan-pink smooth appearance fundus	Atrophic endometrium and adenomyosis, benign cervix
Left pelvic lymph nodes	Lymph node diameter: 0.4-1.5 cm	Tan-yellow fatty tissue with multiple tan lymph nodes of various sizes	13 lymph nodes, benign
Right pelvic lymph nodes	Lymph node diameter: 0.8-1.6 cm	Tan-yellow fatty tissue with multiple tan lymph nodes of various sizes	7 lymph nodes, benign
Left para-aortic lymph nodes	Whole sample: 4.8 cm x 4.0 cm x 1.0 cm	Tan-yellow fatty tissue	3 lymph nodes, benign
Right para-aortic lymph nodes	Lymph node diameter: 0.5-4.8 cm	Tan-yellow fatty tissue with 2 small and one large, possibly matted lymph node	3 lymph nodes, benign
Diaphragmatic tissues	0.5 cm x 0.3 cm x 0.3 cm	Tan tissue fragment	Benign
Peritoneum	Right upper biopsy: 0.6 cm x 0.4 cm x 0.3 cm; right lower biopsy: 0.7 cm x 0.4 cm x 0.3 cm; left upper biopsy: 0.8 cm x 0.6 cm x 0.3 cm; left lower biopsy: 0.8 cm x 0.5 cm x 0.3 cm	Four samples of tan-yellow tissue	Benign
Omentum	13.0 cm x 10.0 cm x 3.0 cm	Multilobulated tan-yellow fatty tissue	Benign
Bladder	0.7 cm x 0.5 cm x 0.3 cm	Tan fragment of bladder	Benign
Cul de sac	0.3 cm x 0.2 cm x 0.2 cm	Tan-yellow tissue fragments with adherent clotting blood measures	Benign

Analysis of the endometrial curettage specimen revealed benign squamous mucosa consistent with atrophic endometrial epithelium. Following confirmation of cancer in the fallopian tubes, the patient consented to the second set of procedures: a total abdominal hysterectomy (TAH), pelvic washing, and nodal dissections to rule out metastatic spread of EOC cells into the peritoneal space. In addition, biopsies of the omentum, bladder, and various connective tissues were performed (Table 2).

Samples from the TAH revealed a uterus with atrophic endometrium and adenomyosis but were negative for metastasis. A cervical biopsy showed only benign changes. The pelvic washing was positive for malignant cells: metastatic high-grade carcinoma. Biopsy of the pelvic and para-aortic lymph nodes found no signs of metastasis. Samples of the bladder, omentum, and other excised tissues were found to be histologically unremarkable.

In the final diagnosis, malignant high-grade serous carcinoma of the fallopian tubes without intraperitoneal metastases was found histologically. The fallopian tube adenocarcinoma was staged as pT1c Nx Mx. Given the positive malignant carcinoma cells found in the pelvic washing, the patient was referred to a medical oncologist, who prescribed and oversaw her chemotherapy. In light of her family history of breast cancer and her BRCA results, the patient opted for a double mastectomy.

## Discussion

As mentioned before, EOC is a histologic diagnosis based on tissue pathology examination after surgical removal. While early detection is essential for increasing the survival rates of patients with EOC, it is rarely picked up in the early stages due to non-specific clinical presentation along with the lack of effective and specific EOC screenings [6].

Current early-stage EOC screening tests have a high incidence of false-negative results, which lead to missed diagnoses, later detection, and overall decreased survival rates. This makes counseling and identification of high-risk patients, such as those with certain BRCA gene mutations, important. Earlier detection of EOC leads to decreased mortality and morbidity [6]. Additionally, family members may also benefit from counseling and genetic testing.

Historically, ovarian cancer originating from the fallopian tubes was thought to be uncommon in the United States, with an age-adjusted incidence of 0.39 per 100,000 women [8]. However, the incidence is now assumed to be much higher. In fact, many high-grade serous carcinomas involving the fallopian tube were previously classified as advanced ovarian carcinoma but are now believed to have actually originated in the fallopian tubes and later spread to the ovaries [8]. This emerging perspective of origin provides healthcare providers and the scientific community a more complete picture of the etiologies and dissemination pattern of ovarian cancer. We hope this will help physicians to have a more extensive knowledge base of such a disease when looking for risk factors and taking care of their patients.

In this case of ovarian cancer, there was a clear fallopian tube origin with insidious progression toward the ovaries. Not only does this case shed more light for providers on the possible origins of such a morbid disease but also reinforces the need for further development of screen tests with more emphasis on fallopian tube monitoring.

## Conclusions

In this case study, a 53-year-old postmenopausal female with a family history of breast cancer presented to the clinic for an annual wellness visit. After much counseling, the patient underwent BRCA genetic mutation testing, and when the results were found to be positive, she underwent preventative bilateral salpingo-oophorectomy. Bilateral serous carcinoma was discovered during pathological analysis, and a further TAH confirmed the fallopian tubes as the origin of her EOC. To reiterate, the main takeaway from this case study is that ovarian cancers can originate outside the ovary; hence, clinicians need to keep a higher index of suspicion to provide the best preventative care to patients when evaluating them for ovarian neoplasms.
